# Rate of Decline of the Oriental White-Backed Vulture Population in India Estimated from a Survey of Diclofenac Residues in Carcasses of Ungulates

**DOI:** 10.1371/journal.pone.0000686

**Published:** 2007-08-01

**Authors:** Rhys E. Green, Mark A. Taggart, Kalu Ram Senacha, Bindu Raghavan, Deborah J. Pain, Yadvendradev Jhala, Richard Cuthbert

**Affiliations:** 1 Conservation Science Group, Department of Zoology, University of Cambridge, Cambridge, United Kingdom; 2 Royal Society for the Protection of Birds, Sandy, United Kingdom; 3 School of Biological Sciences, Department of Plant and Soil Science, University of Aberdeen, Aberdeen, United Kingdom; 4 Instituto de Investigación en Recursos Cinegéticos, Universidad de Castilla–La Mancha, Cuidad Real, Spain; 5 Bombay Natural History Society, Mumbai, India; 6 Wildlife Institute of India, Dehradun, Uttaranchal, India; University of Sydney, Australia

## Abstract

The non-steroidal anti-inflammatory drug diclofenac is a major cause of the rapid declines in the Indian subcontinent of three species of vultures endemic to South Asia. The drug causes kidney failure and death in vultures. Exposure probably arises through vultures feeding on carcasses of domesticated ungulates treated with the drug. However, before the study reported here, it had not been established from field surveys of ungulate carcasses that a sufficient proportion was contaminated to cause the observed declines. We surveyed diclofenac concentrations in samples of liver from carcasses of domesticated ungulates in India in 2004–2005. We estimated the concentration of diclofenac in tissues available to vultures, relative to that in liver, and the proportion of vultures killed after feeding on a carcass with a known level of contamination. We assessed the impact of this mortality on vulture population trend with a population model. We expected levels of diclofenac found in ungulate carcasses in 2004–2005 to cause oriental white-backed vulture population declines of 80–99% per year, depending upon the assumptions used in the model. This compares with an observed rate of decline, from road transect counts, of 48% per year in 2000–2003. The precision of the estimate based upon carcass surveys is low and the two types of estimate were not significantly different. Our analyses indicate that the level of diclofenac contamination found in carcasses of domesticated ungulates in 2004–2005 was sufficient to account for the observed rapid decline of the oriental white-backed vulture in India. The methods we describe could be used again to assess changes in the effect on vulture population trend of diclofenac and similar drugs. In this way, the effectiveness of the recent ban in India on the manufacture and importation of diclofenac for veterinary use could be monitored.

## Introduction

Populations of three species of vultures endemic to South Asia, oriental white-backed vulture *Gyps bengalensis*, long-billed vulture *G. indicus* and slender-billed vulture *G. tenuirostris* have collapsed since the 1990s and are still declining rapidly [1]–[3]. As a result, they are listed as critically endangered [4]. The non-steroidal anti-inflammatory drug (NSAID) diclofenac has been identified as a major cause of the population declines [2], [5], [6]. The drug is widely used in the Indian subcontinent to treat inflammation, fever and pain associated with disease and injury in domesticated ungulates. Oriental white-backed vultures, African white-backed vultures *G. africanus* and Eurasian griffon vultures *G. fulvus* died from kidney failure within a few days of experimental treatment with doses of diclofenac within the range likely to be encountered by birds scavenging tissues from treated ungulates and showed extensive visceral gout at post mortem [5], [7]. Because susceptibility to diclofenac poisoning is widespread within the phylogenetic tree of the genus *Gyps* [8], the two other threatened South Asian species *G. indicus* and *G. tenuirostris* are also likely to be susceptible, though no experiments have yet been performed on them to check this. Visceral gout and diclofenac residues in tissues have been found in most carcasses of wild *Gyps bengalensis* and *G. indicus* from India, Pakistan and Nepal examined since the decline began [5], [6]; the proportion affected being similar to that expected if diclofenac poisoning was the only cause of the vulture decline [2].

The most probable way in which vultures are exposed to diclofenac is by feeding upon carcasses of domesticated ungulates that were treated with the drug shortly before death [2], [9]. However, until the study reported here, no large-scale surveys had been made of the amount of diclofenac in tissues of ungulates available to vultures. Until now, there has also been no method for estimating the effect on vulture population trend of a given distribution of levels of diclofenac contamination in samples taken from ungulate carcasses. Hence, although the Drug Controller General (India) withdrew all licences for the manufacture of diclofenac for veterinary use in 2006, there is no accepted method for monitoring the effectiveness of this ban in terms of its impact on vultures. In this paper, we describe a method designed to fill this gap. We use a survey of diclofenac concentrations in liver samples from carcasses of domesticated ungulates in India, reported in more detail elsewhere [10], to estimate the trend of the oriental white-backed vulture population. We compare these results with the rate of population change measured using repeated counts of vultures on road transects.

## Results

### Outline of the procedure for estimating the rate of vulture population decline from measurements of diclofenac in ungulate liver

We estimated the expected rate of decline of the vulture population using a chain of calculations. The steps involved are described below and are numbered as in Table 1, which is intended as a guide to the logic of the procedure.

**Table 1:** Logical structure of the method used to estimate the rate of population decline of the oriental white-backed vultures in India.

Step	Description
1.	Compare the fit of alternative models of the observed distribution of diclofenac concentrations *d_liver_* in liver samples taken from ungulate carcasses. Select a suitable model with cdf, V(*d_liver_*).
2.	Allow for the component of variation in diclofenac concentration in V(*d_liver_*) attributable to combined sampling and measurement errors by estimating the variation in replicate measurements from different parts of the same liver.
3.	Describe the relationship between the concentration of diclofenac in the liver *d_liver_* and that in other tissues from the same animal. Estimate the concentration of diclofenac averaged across all the edible tissues of a carcass as a proportion of that measured in a sample of liver from the same animal, *a'_tot_*.
4.	From the average meal size and body weight of oriental white-backed vultures, estimate the dose of diclofenac *d_vbw_* ingested per unit vulture body weight from a single meal of mixed tissues from a carcass containing average concentration *d_carc_*.
5.	Fit a dose-response model with cdf J(*d_vbw_*) relating the proportion of oriental white-backed vultures that are killed by taking a single meal from a contaminated carcass to *d_vbw_*.
6.	Combine the results of steps 3, 4 and 5 to obtain a dose-response model with cdf K(*d_liver_*) relating the proportion of oriental white-backed vultures that are killed by taking a single meal of mixed tissues from a contaminated ungulate carcass to the concentration of diclofenac in the liver *d_liver_*.
7.	Integrate the product of the pdf of the distribution of ungulate liver concentration of diclofenac and the cdf of the dose-response model, v(*d_liver_*)K(*d_liver_*), across the distribution of *d_liver_* to estimate the proportion of vultures killed per meal, averaged across all meals taken by the vulture population. This is equivalent to the parameter *C* in the vulture population model of Green *et al*. [2].
8.	Use the value of *C* and the model of Green *et al*. [2] to estimate the expected rate of decline of the vulture population.
9.	Compare the expected rate of decline of the population of oriental white-backed vulture from ungulate carcass sampling in 2004–2005 with the rate estimated from repeated road transect surveys of vulture numbers in a similar area in 2000–2003.
10.	Estimate, from the observed and expected rates of population decline, the proportion of mortality in excess of that expected in a pre-decline stable vulture population that is attributable to diclofenac poisoning.

### Step 1: Fitting a distribution model to the measurements of diclofenac concentrations in ungulate liver samples

**Figure 1:**
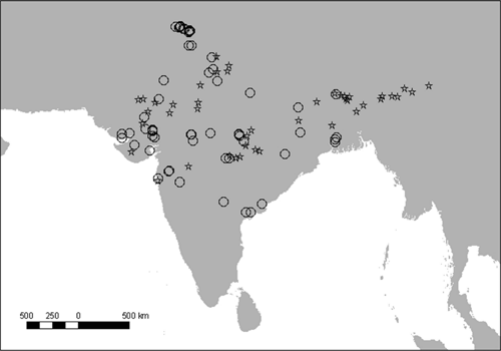
Locations of sites studied in India. Sites from which liver samples were obtained from carcasses of domesticated ungulates in 2004–2005 for diclofenac assays are shown by circles (n = 67) and centroids of 73 road transect surveys used to measure the population trend of the oriental white-backed vulture in 2000–2003 are shown by stars.

**Figure 2:**
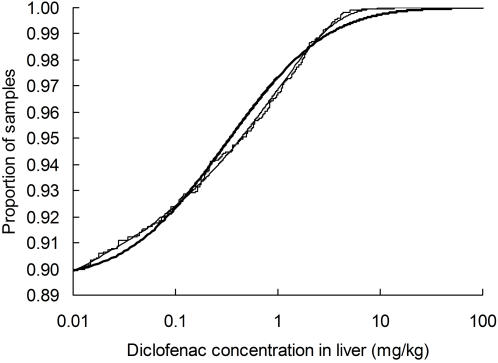
Cumulative distribution of diclofenac concentrations in liver samples. The stepped line shows the observed cumulative distribution of concentrations for 1,848 liver samples. Also shown is the fitted cumulative log-normal distribution in which the mean of log_e_-transformed values is −1.1522 and standard deviation is 1.7670 (thick curve). The thin curve is the fitted third order complementary log-log model in which the cumulative probability is 0.8765+0.1235 (1–exp(-exp(0.3184+0.5415 log_e_(*d_liver_*)+0.05110 (log_e_ (*d_liver_*))^2^+0.005058 (log_e_(d_liver_))*^3^*)).

We assumed that the livers of a proportion *f* of total sample of *N* carcasses contained residues of diclofenac and that the remainder (1-*f*) had no trace of the drug. Diclofenac concentrations *d_liver_* of those samples with traces of the drug present were assumed to be distributed according to some function with cumulative distribution function (cdf) U(*d_liver_*). Hence, the cdf of the distribution of diclofenac concentration in all carcasses was taken to be V(*d_liver_*) = 1+*f* (U(*d_liver_*)−1). Diclofenac concentrations less than the LOQ (0.01 mg kg^−1^) could not be measured and the left-hand side of the distribution of *d_liver_* was therefore veiled. Hence, we fitted truncated (left-censored) versions of U(*d_liver_*) to the *n* observations with measurable diclofenac from our 67 sampling sites (Fig. 1), using a maximum-likelihood method [11] and the NONLIN module of SYSTAT 5.03. We compared the fit of truncated forms of two types of distribution to the >LOQ measurements of diclofenac in liver; the log-normal distribution and the complementary log-log distribution U(*d_liver_*) = 1-exp(-exp(z(*d_liver_*)), where z(*d_liver_*) is a *m*th order polynomial, *b_0_*+*b_1_* log_e_(*d_liver_*)+*b_2_* (log_e_ (*d_liver_*))^2^…+*b_m_* (log_e_(d_liver_))*^m^*. Having fitted each of these distributions, we estimated *f* = *n*/*N*(1−U(0.01)). None of the distributions gave a significantly poor fit, according to the one-sample Kolmogorov-Smirnov test (Table 2). However, the log-normal distribution fitted the data significantly less well than any of the complementary log-log models (likelihood-ratio tests, *P*<0.05). The second order complementary log-log distributions gave the lowest value of the Akaike Information Criterion (AIC), but the third order model had a similar AIC value (Table 2). We selected the third order model for use in later steps of the procedure because it gave the lowest value of Kolmogorov-Smirnov's D. Comparison of a cumulative plot of the data with the fitted log-normal and third order complementary log-log distribution illustrates the relatively good fit of the latter model (Fig. 2). Measurable levels of diclofenac were found in 10% of the samples and the concentration exceeded 1 mg kg^−1^ in 3.3% of samples. The proportion of samples with diclofenac varied to a limited extent geographically and according to the type of sampling sites and ungulate species, age and sex, but these effects are examined in detail in another paper [10].

**Table 2:** Performance of models of the distribution of diclofenac concentrations measured in samples of liver tissue.

Model	Residual deviance	Number of parameters	AIC	Kolmogorov-Smirnov D	K-S *P*
log-normal	2888.96	2	2892.96	0.085	0.13
C L-L first order	2870.14	2	2874.14	0.054	>0.40
C L-L second order	2867.22	3	2873.22	0.048	>0.40
C L-L third order	2865.34	4	2873.34	0.042	>0.40
C L-L fourth order	2863.67	5	2873.67	0.045	>0.40

Only samples with concentrations above the LOQ (0.01 mg kg^−1^) were included and all fitted distributions were truncated at the LOQ. The residual deviance, number of fitted parameters, Akaike Information Criterion (AIC) and the maximum difference between observed and fitted cumulative distributions (D from the Kolmogorov-Smirnov one sample test) are shown for each model. For the complementary log-log (C L-L) models, results are shown for first to fourth order polynomials.

To assess the effects of sampling error on the fitted values of the parameters of complementary log-log distribution, for use in later steps of the procedure, we generated 10,000 associated sets of estimates of *f*, *b_0_*, *b_1_*, *b_2_* and *b_3_* by a bootstrap procedure. We considered using sampling sites as units for bootstrapping. However, 10 samples or fewer were collected at 42 of the 67 sites, whilst more than 100 samples were collected at 7 sites. For this reason, we grouped the sites into 21 clusters with less variable combined sample size by pooling data from neighbouring sites so that each cluster included at least 25 samples. We then obtained confidence intervals by drawing 10,000 bootstrap samples with replacement, using clusters as the sampling units. Each bootstrap sample consisted of data for all samples from 21 clusters drawn at random, with replacement, from the 21 clusters in the actual dataset. The distribution model was then fitted to each bootstrap sample, as described above, and the associated sets of parameter values were saved for use in subsequent stages of the analysis.

### Step 2: Allowing for effects of within-liver sampling and measurement errors on the variance of observed concentrations of diclofenac in liver

Lack of precision in our measurements of diclofenac concentration in liver would increase the apparent variation among samples described by the functions U(*d_liver_*) and V(*d_liver_*). If the error was sufficiently large, this difference in variance would affect our estimates of the average proportion of vultures killed per meal in Step 7, even if the proportion of samples contaminated and the mean concentration remained the same. Hence, we performed a one-way ANOVA to partition the variance of measurements of diclofenac in six replicated liver samples from each of five cattle into among-cow and within-cow components. Concentrations were log_e_-transformed before analysis. We took the error mean square as the variance of replicate measurements of the same liver.

The error mean square was 0.07228 (error sum of squares = 1.807, error d.f. = 25). This is equivalent to a coefficient of variation due to combined measurement error and within-liver sampling error of 31% (exp(√0.07228) = 1.3085). This level of variation is small relative to that observed among livers from different animals in our larger sample. The log-normal distribution fitted in Step 1 has a mean of −1.1522 and a standard deviation of 1.7670. Hence, the proportion of the variance described by the log-normal form of U(*d_liver_*) that is attributable to within-liver sampling error and measurement error is just 2% (0.07228/(1.7670^2^)). The standard deviation of the log-normal distribution after allowing for within-liver sampling error and measurement error is 1.7464 (i.e. √(1.7670^2^−0.07228)), which is sufficiently similar to the unadjusted value of 1.7670 that we expect that adjustment would have a negligible effect on the estimate of vulture population trend. Therefore, we ignore this effect in subsequent steps. We do this because, although we could readily make the adjustment to the log-normal form of U(*d_liver_*) and V(*d_liver_*), it is not straightforward to do so when using the complementary log-log distribution, which we selected over the log-normal in Step 1 because of its superior fit to the data. We check on the consequences of this decision in Step 7.

### Step 3: Estimating the average concentration of diclofenac in the whole carcass from the observed value for liver

**Figure 3:**
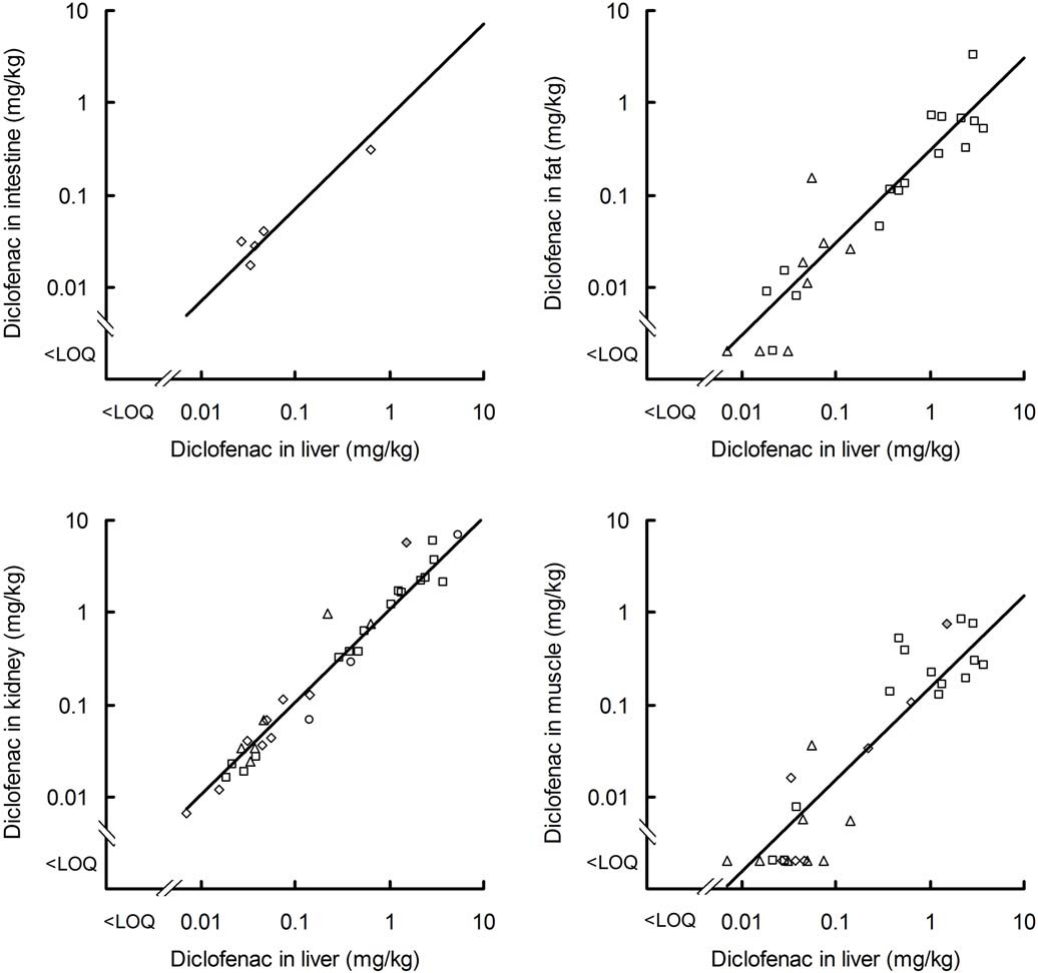
Relationship of diclofenac concentration in selected tissues to that in liver from the same ungulate. Log-log plots are shown of the concentration of diclofenac in intestine, fat, kidney and muscle against that in the liver. Symbols denote ungulate species and data source: open diamonds-*Bos indicus*, Experiment 1 [9]; circles-*Bos indicus*, Indian carcass dumps; squares-*Bos taurus*, Experiment 2; triangles-*Bos taurus*, Experiment 3; grey diamond–*Bubalus bubalis*, [5]. Lines show results from the fitted Model E, in which the geometric mean concentration in the selected tissue is assumed to be a fixed proportion of the concentration in liver (*k* = 1, see text).

We wished to estimate the concentration of diclofenac, averaged across the edible parts of an ungulate carcass available to vultures, relative to that in the liver of the same animal. Because vultures usually eat virtually all the soft tissues, knowing this conversion factor allows us to calculate the average diclofenac concentration in the food that vultures take from a carcass from that in the liver. We first modelled the relationship between the diclofenac concentration in a given tissue *d* and that in the liver of the same animal *d_liver_*, using diclofenac analyses of paired samples of liver and other tissues. Only data for which the concentration in liver was above the limit of quantification (LOQ) were included in the analysis. Inspection of log-log plots suggested that these relationships were similar for different species and sample sources (Fig. 3), so we ignored species and source in our analyses. We fitted the model *d* = *a d_liver_^k^*, where *a* and *k* are constants estimated from the data, using a quasi-Newton maximum-likelihood (M-L) method in the NONLIN module of SYSTAT 5.03.

We assumed that the differences between the natural logarithms of observed and modelled values were normally distributed with standard deviation *v*, which we also estimated. The M-L procedure was preferred to ordinary linear least squares regression after log transformation of *d* because some concentrations in tissues other than liver were below the LOQ. The presence of <LOQ observations was handled in the M-L procedure by incorporating into the model left-censoring of *d* at the appropriate LOQ value [11], which varied according to the experiment and tissue [5], [9].

The formulation of our model allows for the possibility that the concentration of diclofenac in a given tissue, as a proportion of that in liver, might not be a constant, but may vary with *d_liver_*. Only if *k* = 1, would the expected value of *d* be a fixed proportion of *d_liver_*.

We made different assumptions about how *a, k* and *v* vary among tissues in different models. Model fit was assessed using residual deviance and AIC. Assuming that parameters *a* or *v* had the same value for all tissues resulted in models with relatively poor fit (Models B and D compared with Model A; Table 3), so we fitted these parameters separately for each tissue. Assuming that *k* had the same value for all tissues did not result in markedly poorer fit than when it was estimated separately for all four tissues (Model C compared with Model A). The estimated common value of *k* from Model C was 1.0432, which suggested that taking *k* = 1 would be a reasonable further simplification. Model E, which has tissue-specific values of *a* and *v* and *k* = 1, performs similarly to Models C and A and has the lowest AIC value of all the models we assessed. Hence, we prefer this model, which treats the concentration of diclofenac in a tissue as a fixed proportion of that in the liver.

**Table 3:** Comparison of five models of the relationship between diclofenac concentration in a specified tissue and the concentration in liver from the same animal.

Model	Model specification			Residual deviance	Number of parameters	AIC
	*a*	*k*	*v*			
A	T	T	T	1227.33	12	1251.33
B	C	T	T	1281.24	9	1299.24
C	T	C	T	1232.12	9	1250.12
D	T	T	C	1248.36	9	1266.36
E	T	1	T	1233.63	8	1249.63

The parameters *a, k* and *v* (see text) are either assumed to vary among tissues (T) or to have a common value across all four tissues (C). In Model E the parameter *k* is set to the value 1 for all tissues. The residual deviance, number of fitted parameters and Akaike Information Criterion (AIC) are shown for each model. Models with the lowest AIC have reasonable fit without requiring the estimation of many parameters.

The value *a* for a given tissue, is the geometric mean of the relative concentration of diclofenac in that tissue, as a proportion of that for liver from the same animal. To convert observed liver concentrations from carcasses into estimates of diclofenac concentration in each tissue, we require estimates of the arithmetic mean relative concentration *a'*. We calculated these by the method proposed by Rothery [12], as *a'* = exp(log_e_(*a*)+0.5*v* ^2^). Relative concentrations calculated in this way for intestine, fat, kidney and muscle are shown in Table 4. The average concentration of diclofenac across all of the edible parts of the carcass *a'_tot_*, relative to that of liver, was calculated from these relative concentrations in each tissue and the composition of the carcass (Table 4). The average carcass composition of cattle and water buffalo were taken from ref. 9. We used a Monte Carlo procedure to obtain 95% confidence limits for *a'_tot_*. We generated 10,000 pairs of random normal deviates for each tissue and used them and the variance-covariance matrix provided by SYSTAT to calculate 10,000 associated pairs of values of *a* and *v* for each tissue. For each replicate, we then calculated *a'_tot_*, as described above. We ranked the 10,000 *a'_tot_* values and took the bounds of the central 9,500 values as the 95% confidence limits.

**Table 4:** Arithmetic mean concentration of diclofenac in tissues, as a proportion of that in liver from the same animal *a'* (see text).

Tissue(s)	Relative concentration	95% C.L.	Proportion of carcass
Intestine	0.7446	0.2476–2.1596	0.1681
Fat	0.3984	0.1144–1.2984	0.2001
Kidney	1.1772	0.6492–2.1455	0.0056
Liver	1.0000	n/a	0.0208
Muscle	0.2372	0.0500–1.3003	0.6054
Edible carcass	0.3759	0.2042–1.0717	1.0000

The arithmetic mean concentration of diclofenac averaged across the edible parts of the entire carcass *a'_tot_* is also shown. This was calculated by assuming that the proportion of the carcass composed of the different tissues is as indicated in the right hand column. Monte Carlo 95% confidence limits for the relative concentrations are shown.

The concentration of diclofenac was highest in kidney and lowest in muscle (Table 4). Averaging across all soft tissues, the arithmetic mean concentration of diclofenac in the whole of the edible carcass was 38% of that in the liver.

### Step 4: Relationship between diclofenac concentration in ungulate liver and the dose per unit vulture body weight ingested by oriental white-backed vultures

Following ref. 13, we assumed that a free-living wild oriental white-backed vulture requires an average daily food intake 0.341 kg of ungulate tissue. Hence, the amount of food ingested per meal is 0.341 *F* kg for a vulture feeding at intervals of *F* days. A vulture feeding on an average mixture of edible tissues from a carcass with a liver diclofenac concentration *d_liver_* would ingest 0.341 *a'_tot_ F d_liver_* mg of diclofenac per meal. Given that the average weight of an oriental white-backed vulture is 4.75 kg [13], the dose of diclofenac ingested per unit vulture body weight *d_vbw_* is given by (0.341 *a'_tot_ F d_liver_*)/4.75.

### Step 5: Model of dose-dependent mortality of oriental white-backed vultures

**Figure 4:**
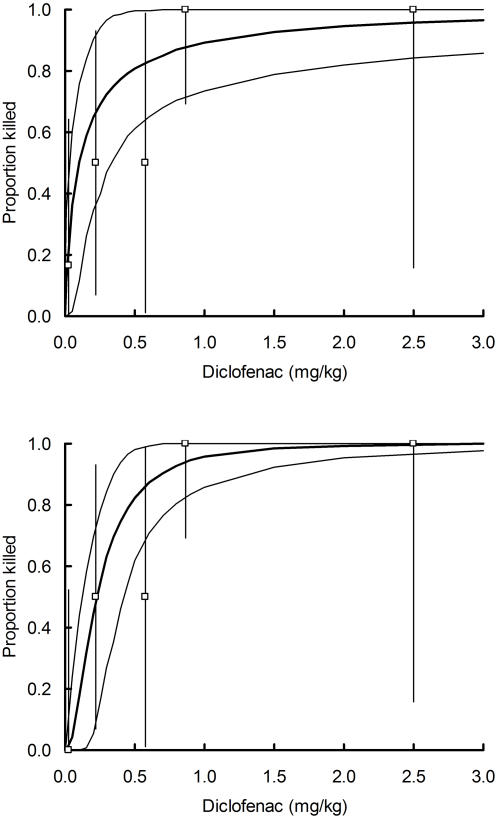
Relationship between the proportion of oriental white-backed vultures treated experimentally with diclofenac that died and the dose of diclofenac administered per unit vulture body weight. Plotted points are proportions killed for each of five bins of dose, with 95% exact binomial confidence limits (vertical lines). Bins include 6, 4, 2, 10, and 2 birds respectively (ranked from lowest to highest dose). The thick curve is the fitted probit model relating mortality rate to log dose. Thin curves show the envelope enclosing the central 9,500 of 10,000 Monte Carlo replicate values. The upper panel shows results of analysis of all data. The lower panel excludes the datum for an outlier (vulture Gb11) from the lowest dose bin, which died even though it apparently received an extremely low dose of diclofenac. Data are from Table 2 of Oaks *et al.* [5].

We used experimental data for captive oriental white-backed vultures from ref. 5 to fit a probit dose-response model relating the proportion of birds killed by diclofenac to the dose of the drug administered per unit vulture body weight. We assumed that the natural logarithm of the lethal dose of diclofenac in mg kg^−1^ of vulture body weight is specified by a normal distribution with mean *m* and standard deviation *s*. Hence, the cdf of this normal distribution J(*d_vbw_*) describes the probability of death after eating a contaminated meal that results in a dose per unit vulture body weight *d_vbw_*. Models were fitted by a maximum-likelihood method using the NONLIN module of SYSTAT 5.03. We used two versions of the model, which were fitted with and without data from an outlier; a vulture coded Gb11 which died with visceral gout after apparently receiving a very low dose of diclofenac (see ref. 7). We calculated confidence limits using the same Monte Carlo procedure as described in Step 3. We took the bounds of the central 9,500 values as the 95% confidence limits. The fitted mean *m* and standard deviation *s* of the natural logarithm of the lethal dose of diclofenac in mg kg^−1^ of vulture body weight were −2.3273 (95% confidence limits −3.5715 to −1.0865) and 1.8870 (0.5807 to 3.0795) respectively with Gb11 included and −1.4934 (−2.1274 to −0.86123) and 0.8675 (0.3348 to 1.3744) respectively with Gb11 excluded. The two versions of the model are illustrated in Fig. 4.

### Step 6: Vulture mortality rate per meal in relation to diclofenac concentration in the liver of the ungulate eaten and the interval between meals

Steps 3, 4 and 5 were used in combination to specify the relationship between the probability of death of a vulture per meal and the concentration of diclofenac in the liver of the ungulate from which a meal of mixed tissues was taken. This relationship is the cdf of a normal distribution K(*d_liver_*) with standard deviation *s*, from Step 5, and a mean given by *m*-log_e_(0.341 *a'_tot_ F d_liver_*/4.75), where *a'_tot_* is from Step 3 and *m* is from Step 5. The back-transformed median lethal dose in liver from this calculation, for vultures feeding on the whole carcass, was 1.81 mg kg^−1^ (for feeding interval *F* = 2) or 1.21 mg kg^−1^ (for *F* = 3), if the outlier Gb11 was used in fitting the dose-response model, and 4.16 mg kg^−1^ and 2.77 mg kg^−1^ respectively if Gb11 was excluded.

### Step 7: Average vulture death rate per meal

The average proportion of vultures dying from diclofenac poisoning per meal if they feed from carcasses with a distribution of diclofenac concentrations in liver with cdf V(*d_liver_*) is given by the integral, with respect to *d_liver_*, of the product v(*d_liver_*)K(*d_liver_*), for a given interval *F* between meals, where v(*d_liver_*) is the probability density function (pdf). This death rate per meal *C* is equivalent to the parameter *C* in the vulture population model of Green *et al*. [2]. In that paper, *C* was defined as the proportion of carcasses that contained sufficient diclofenac to cause the death of all vultures that fed. We obtained confidence limits for *C* by repeating all the calculations described above using each of the 10,000 bootstrap or Monte Carlo replicate estimates of the parameter sets for each step and taking the central 9,500 values of the resulting estimates of *C* as 95% confidence limits. The estimated death rate per meal was 2.66% (for feeding interval *F* = 2) or 3.23% (for *F* = 3) if the outlier Gb11 was used in fitting the dose-response model, and 0.86% and 1.37% if Gb11 was excluded (Table 5).

**Table 5:** Estimates of death rate per meal *C* of oriental white-backed vultures, the annual rate of decline of the vulture population and the percentage of excess vulture mortality that is attributable to diclofenac poisoning *E*, based upon concentrations of diclofenac in liver samples taken from ungulate carcasses.

Model specification			Death rate per meal *C* %		Annual rate of decline %			% of excess mortality caused by diclofenac *E_diclo_*		Percent support for diclofenac as	
Gb11 included?	*F*	*S_0_*	Estimate	95% C.L.	Estimate	95% C.L.	Different from road transects? *P*	Estimate	95% C.L.	Main cause of decline	Sole cause of decline
Yes	2	0.90	2.66	0.40–5.72	99	57–100	0.018	100	100–100	99.4	98.2
Yes	2	0.97	2.66	0.40–5.72	99	53–100	0.021	100	100–100	99.5	97.9
Yes	3	0.90	3.23	0.56–6.45	98	54–100	0.014	100	100–100	99.6	98.6
Yes	3	0.97	3.23	0.56–6.45	98	51–100	0.016	100	100–100	99.5	98.4
No	2	0.90	0.86	0.07–3.11	81	19–100	0.136	100	19–100	93.7	86.4
No	2	0.97	0.86	0.07–3.11	80	14–100	0.156	100	17–100	92.9	84.4
No	3	0.90	1.37	0.19–4.05	83	28–99	0.100	100	40–100	95.9	90.0
No	3	0.97	1.37	0.19–4.05	82	23–99	0.114	100	35–100	95.4	88.6

A significance test of the difference between the rate of population decline estimated from diclofenac surveys and road transect counts of vultures and the level of support (% of bootstrap/Monte Carlo replicates) for diclofenac poisoning being the main cause (>50% of excess mortality) or the sole cause (100% of excess mortality) of the vulture decline are also shown. Results are given for calculations that used a dose-response model including and excluding an outlying datum (Gb11), for two plausible values of the interval between meals *F* (days) and for the bounds of the likely range of pre-decline annual adult survival probability *S_0_*.

Although the log-normal model of the distribution of diclofenac values did not fit the data particularly well, it gave estimates for the death rate per meal that were similar to those for the complementary log-log distribution. For the log-normal, *C* was 2.59% (for *F* = 2) or 3.13% (for *F* = 3) if the outlier Gb11 was used in fitting the dose-response model, and 0.99% or 1.40% if Gb11 was excluded. Using the version of the log-normal distribution with its standard deviation adjusted to allow for the effect of combined within-liver sampling and measurement error (see Step 2) gave very similar results to those from the unadjusted log-normal (2.58, 3.12, 0.97 and 1.38% respectively for the four values cited earlier). Hence, our decision in Step 2 to ignore within-liver sampling error and measurement error as having a negligible effect on subsequent calculations is justified.

### Step 8: Vulture population trend estimated from diclofenac concentrations in ungulate liver samples

We used the vulture population model from ref. 2 to estimate the expected annual rate of change of the oriental white-backed vulture population in 2004–2005 from the *C* values derived from observed levels of diclofenac contamination of ungulate carcasses in our sample. Following ref. 2, we took 0.90 and 0.97 as the bounds of the likely range for the pre-decline annual adult survival rate *S_0_* and used values of 2 and 3 days as the interval between meals *F*. Values of the other parameters in the model are as used by Green *et al*. [2]. Confidence intervals were obtained as in Step 7. We expect high rates of population decline (>98% per year) when the dose-response model includes the outlier Gb11, whereas lower rates of decline (80–83% per year) are expected when Gb11 is excluded (Table 5).

### Step 9: Population trends of the oriental white-backed vulture in India from road transect counts

We estimated the average population trend from transect counts in 2000–2003 by fitting a log-linear Poisson regression model in GLIM with vulture count as the dependent variable, transect modelled as a factor and years elapsed since 2000 as a covariate [2]. The annual rate of population decline, as a percentage, was calculated as 100 (1–exp(*g*)), where *g* is the regression coefficient for count on elapsed years from the fitted regression model. Confidence intervals were obtained by drawing 10,000 bootstrap samples, using transects as the sampling units for bootstrapping purposes. Each bootstrap sample consisted of data from all years for 73 transects drawn at random, with replacement, from the 73 eligible transects in the real dataset. The regression model was then fitted to each bootstrap sample, as described above, and the central 9,500 estimates were taken to define the 95% confidence limits of the rate of population decline.

The average rate of change of oriental white-backed vultures, averaged over the period 2000–2003 was a decline of 48% per year (95% confidence limits 34–62%). This is a much lower rate of decline than those (98–99% per year) calculated from diclofenac levels in carcasses in Step 8 using the dose-response model that included the datum from the outlier Gb11. We tested the significance of the difference between population trend estimates from the carcass suvey and road transects by using as the *P* value the proportion of bootstrap/Monte Carlo replicates in which the difference between the two types of estimate was of opposite sign to that from the point estimates. This indicated that the rate of decline derived from carcass surveys was significantly higher (*P*<0.021; Table 5). However, when the dose-response model fitted after excluding Gb11 is used in Step 8, the rates of decline obtained from carcass surveys were more similar (80–83% decline per year) to the rate calculated from road transect data and there was no significant difference between the two types of estimate (*P*>0.10; Table 5).

### Step 10: Was diclofenac poisoning the main or sole cause of the vulture population decline?

We assumed that the vulture population decline might have been caused by a combination of diclofenac poisoning and some other unknown factors that were not operating, at least to the same extent, before the decline started, such that the additional daily mortality rate for diclofenac poisoning and the unknown factors together is *A*. We call *A* the excess mortality. We estimated *A* from the rate of vulture population decline measured using the road transect data (Step 9) for a specified value of *S_0_* and *F* = 1, using the method used by Green *et al.* [2] to estimate the value of *C* required to produce the observed declines. We then calculated the percentage of excess mortality caused by diclofenac as *E_diclo_* = 100 log_e_(1-*C*)/(*F* log_e_(1-*A*)), using the estimates of *C* for values of *F* and other conditions set out in Step 8. *E_diclo_* was constrained to equal 100 when the estimate exceeded 100. Confidence intervals were calculated as in steps 7 and 8. We took the proportion of bootstrap/Monte Carlo replicates in which *E_diclo_*>50 to indicate the level of support for diclofenac being the main cause of the vulture decline and the proportion with *E_diclo_* = 100 as the level of support for diclofenac being the sole cause of the decline.

For all versions of the population model examined, our best estimate is that diclofenac poisoning was the sole cause of the decline (Table 5). After taking into account the various sources of uncertainty in the estimates, the level of support for this hypothesis exceeded 84%. Support for the hypothesis that diclofenac poisoning was the main cause of the decline exceeded 92%.

## Discussion

Our results indicate that the level of diclofenac contamination found in carcasses of domesticated ungulates available to vultures in India in 2004–2005 was sufficient to account for the observed decline of the oriental white-backed vulture population, measured using road transect data in 2000–2003. The hypothesis that an unknown major cause of mortality, in addition to diclofenac, contributed to the vulture declines is not supported by our analysis because the estimated rate of population decline from the carcass surveys was higher than that estimated from road transects counts. This difference was statistically significant if the outlier in the dose-response data was included, but not if the datum for this bird was excluded.

Although the difference was not statistically different if the outlier was excluded, we estimated a substantially more rapid rate of population decline from ungulate carcass surveys than from vulture counts. There are several reasons to expect a real difference in this direction. The pattern of geographical variation in vulture abundance in recent years is likely to have been different from that of diclofenac contamination. If vultures declined to a greater extent prior to 2000 in areas with high levels of diclofenac use, this would lead to lower exposure and rate of population decline in 2000–2003 than if vultures were uniformly distributed across our study area, as is assumed by our method. Furthermore, carcass sampling began more than a year after the road transect surveys ended. The use of diclofenac, and hence carcass contamination, may well have increased during this time and this would make the rate of population decline estimated from carcass surveys appear higher than that from road transects.

A further reason to expect a higher rate of decline from our ungulate carcass surveys than from counts is the source of the relationship we used between vulture mortality and diclofenac dose. We used information from experiments conducted in Pakistan on birds taken into captivity within four years of the first use of diclofenac as a veterinary medicine in that country [3], [5]. However, the transect count estimate of vulture population trend in India used counts made in 2003–2004, which is 6–9 years after the probable first veterinary use of diclofenac in India in about 1994. The oriental white-backed vulture population in India is estimated to have declined by 96% by 2000 [1], so there is likely to have been strong selection, acting over a longer period than in Pakistan, for those vultures least likely to succumb to diclofenac poisoning. The effect of using a dose-response relationship derived from experiments on birds from a population with only short-term exposure (Pakistan) to diclofenac to estimate impact on a population exposed for a longer period (India) may have been to overestimate both mortality from a given dose and decline rate.

Finally, our estimates ignore the fact that vultures feed on carcasses of wild as well as domesticated ungulates. Because the former do not contain diclofenac residues, this will lead us to overestimate death rate per meal and population decline rate. However, there are very large numbers of domesticated ungulates in India, relative to those of wild ungulates. Even in the Gir Forest (Gujarat, India), an area with above average densities of wild ungulates, 93% of ungulate carcasses available to vultures were of domesticated species [14]. Hence, we think that the error caused by this omission is unlikely to be large.

A weakness of our study is that we cannot be certain that our samples of ungulate carcasses were representative of those at which oriental white-backed vultures obtained their food during the period over which their rate of population decline was measured. This might lead to bias in either direction. Given the difficulty of defining a population of potential vulture foraging sites from which a random sample might be drawn, the current rarity of vultures in India and the lack of previous quantitative studies of the relative use of different types of foraging sites, we cannot see how this deficiency could be overcome. Our sampling sites were of types for which there is anecdotal evidence of vultures obtaining food, were widely distributed geographically, and there was no obvious way in which sampling was biased towards locations or animals with an atypically high probability of diclofenac treatment. Hence, we think that there is unlikely to be substantial bias in our estimates for this reason.

The relationship of vulture mortality from kidney failure to the dose of diclofenac ingested differed markedly according to whether or not the datum from a single outlier from the experiments reported by Oaks *et al.* [5] was included in the calculation. This had a large effect on the estimate of the expected rate of vulture population decline, which was much more rapid if the outlier was included. For reasons considered in detail by Swan *et al.* [7], it is not clear whether this observation should be included or not. However, our conclusion about whether the level of diclofenac contamination of ungulate carcasses is sufficient to account for the vulture decline does not depend upon which version of the dose-response model is used.

The low precision of our estimate of vulture population trend reduces the practical value of our method for monitoring the likely effect on vultures of future changes in diclofenac prevalence to some extent. However, analyses of the sensitivity of the precision of the population trend estimate to the precision of the estimates of parameters used in its calculation (not shown) indicate that lack of precision in defining the relationship between vulture mortality and dose made a large contribution to the low precision of the trend estimate. Captive populations of oriental white-backed vultures are now too small and essential for conservation breeding programmes for any new lethal experiments to be performed to refine the estimates defining the dose-response relationship. Nonetheless, if it is assumed that the dose-response relationship will change relatively slowly over time, then the bias introduced by error in the dose-response estimates can be taken to be similar in each repeated future trend estimate. This argument also applies to several of the other steps in our calculation. Hence, our method can be used to estimate changes in population trend with considerably better precision than that of the individual point estimates.

Our conclusions resemble those of a study that used completely different methods. Gilbert *et al*. [3], working in Pakistan, used clusters of oriental white-backed vulture deaths in time and space to estimate the number of point sources of high exposure, probably corresponding to carcasses containing lethal levels of diclofenac, encountered per unit time by vultures from a colony. This rate was combined with an estimate of the number of carcasses consumed by the colony to calculate the proportion of carcasses used as food that contained lethal levels of diclofenac. The range of proportions of lethally contaminated carcasses estimated for the three colonies studied was 1.4–3.0%, which exceeds the 0.4–0.7% of contaminated carcasses calculated by Green *et al*. [2] as being needed to cause the observed rate of decline of this vulture population. This four-fold difference is probably due to an important difference between the two studies in the definition of a lethally contaminated carcass. The clustering method used by Gilbert *et al*. detects any carcass that caused the death of a substantial number of vultures, but such a carcass need not necessarily kill all the birds that fed from it. Only 40–80% of vultures are likely to be killed by taking a large meal (1.023 kg) from the carcass of a cow given a standard veterinary dose of diclofenac immediately before its death [9]. Hence, it seems unlikely that many of the contaminated carcasses detected by Gilbert *et al*. killed even the majority of the vultures that fed from them. The effect of a contaminated carcass in their study would therefore be substantially less than that assumed in the model of Green *et al*. [2], who defined a lethally contaminated carcass as one which killed all the vultures that fed from it: all other carcasses being assumed to kill no vultures. Hence, both our present study in India and that of Gilbert *et al*. in Pakistan indicate that the level of diclofenac contamination of ungulate carcasses is broadly similar to that expected if diclofenac poisoning was the sole cause of the declines.

In 2006, legal measures were introduced by the Government of India to prevent the manufacture and importation of diclofenac for veterinary use and hence reduce the exposure of wild vultures to the drug. Similar steps are being taken in Nepal and Pakistan. Our study relates to the situation in 2004–2005 before this ban was introduced. If the ban results in a rapid decline to zero in the prevalence of diclofenac in ungulate carcasses then this study will prove to have been largely superfluous because there will be no possibility of a continuing negative impact of diclofenac on vultures. However, such a rapid resolution of this problem seems unlikely for several reasons. The ban applies to manufacture and importation for veterinary use, but not for use in human medicine, which is widespread. It is probable that formulations intended for humans will be used to treat ungulates. The ban does not cover the sale of existing stocks of veterinary diclofenac, the magnitude of which is not known. There may also be illegal manufacture and, more probably, illegal importation. Finally, manufacture and importation of other NSAIDs toxic to birds have not been banned and it is possible that the use of these may increase and pose a threat to vultures [15]. Given these possible obstacles to the rapid removal of toxic NSAIDs from the food supply of vultures, we suggest that the methods and results reported in this paper may prove to be valuable in future. They provide a basis for estimating the likely vulture population trend from surveys of diclofenac prevalence, even in areas where vultures have disappeared or declined to such low levels that meaningful estimates of trend cannot be obtained from counts. They also provide a baseline prior to the ban with which future assessments can be compared. We recommend that surveys of contamination of ungulate carcasses with diclofenac and other NSAIDs be undertaken at regular intervals and analyses made of the likely impact on vulture populations. Such a programme will establish whether the legal measures and other actions, such as encouragement of the use of the alternative NSAID meloxicam [16], have diminished the risk to vultures sufficiently to allow wild populations to recover or successful re-introductions to be made using captive stocks.

## Materials and Methods

### Sampling of livers from domesticated ungulate carcasses and measurement of diclofenac concentration

Between May 2004 and June 2005, liver samples from 1,848 carcasses of domesticated ungulates (*Bos indicus, B. taurus,. Bubalus bubalis, Ovis aries, Capra hircus, Equus caballus* and *Camelus* sp.) were collected from 67 sites (Fig. 1). Most sampling sites were carcass dumps managed by local government corporations, co-operatives and private companies or individuals and cattle welfare charities, but 15% of samples were collected from slaughterhouses. The latter were included in the survey because, although some of the meat from slaughtered animals is consumed by humans, a substantial quantity of offal and poor quality meat is disposed of on carcass dumps and therefore becomes available to vultures. A few carcasses (*n* = 7) were found singly in the countryside and alongside roads.

Samples were gathered opportunistically where it was possible to obtain access and permission easily. Hence, although the sites sampled were not necessarily a representative sample of all locations at which tissue from domesticated ungulates was available to vultures, we did not consciously select sites based on any criteria that we believe are likely to lead to an atypical prevalence of diclofenac-treated animals. At all sites except one, every carcass that arrived during the visit was sampled. Hence, there was no possibility of bias within these sites with respect to the species, age, sex or condition of the dead animals sampled. At one site, where 61 samples were obtained, the large numbers of carcasses arriving did not permit all to be sampled and young, prime and mature adults were selected.

Representative samples of liver were taken and temporarily stored on ice prior to freezing. Diclofenac was extracted with acetonitrile and its concentration determined by LC-ESI/MS (liquid chromatography-electrospray ionisation mass spectrometry). ). The limit of quantification (LOQ) for this technique (back calculated to wet tissue concentration) was 0.01 mg kg^−1^. Full details of sample collection, species, age and sex composition, storage, analysis protocols and the precision of estimates are given elsewhere [10].

### Diclofenac concentration in ungulate tissues relative to that in the liver of the same animal

The data for these analyses came from previous studies in which samples of liver and other tissues were available from the same animal. Diclofenac measurements from intestine, kidney and muscle (5, 6 and 6 animals respectively) paired with those for liver samples from the same Indian humped cattle were taken from Experiment 1 of Green *et al.* [9]. Measurements for fat, kidney and muscle paired with those for liver samples from European cattle taken from Experiment 2 (16, 16 and 14 animals respectively) and Experiment 3 (8 animals for each tissue) of ref. 9. We also used measurements for kidney paired with those for liver samples from three Indian humped cattle from carcass dumps in India and kidney and muscle samples from a domesticated water buffalo analysed by Oaks *et al*. [5]. Except for the carcass dump animals, all samples came from ungulates injected with diclofenac experimentally. Details of the sources of the samples from experiments and methods of diclofenac analysis are given elsewhere [5], [9].

### Population trends of the oriental white-backed vulture in India

We used information drawn from 397 vulture counts made in 2000, 2002 and 2003 along 155 road transects distributed widely in India, apart from the southern Deccan peninsula, and surveyed in at least two years (93, 155 and 149 transects totalling 11,183, 18,978 and 18,553 km in road length surveyed in 2000, 2002 and 2003 respectively). Methods and transects are described elsewhere [1], although the network of transects was subsequently expanded. Transects on which no birds were recorded in any year did not contribute information to the estimate of trend, so only the 73 transects on which oriental white-backed vultures were recorded in at least one year were selected for further analysis (Fig. 1)
